# Stem Cells and Liver Diseases

**DOI:** 10.1155/2019/9271746

**Published:** 2019-06-20

**Authors:** Zongyi Hu, Yuchen Xia, So Gun Hong, Emmanuel Thomas, Dali Li

**Affiliations:** ^1^National Institute of Diabetes and Digestive and Kidney Diseases, Bethesda, USA; ^2^State Key Laboratory of Virology, School of Basic Medical Sciences, Wuhan University, Wuhan, China; ^3^National Heart, Lung, and Blood Institute, Bethesda, USA; ^4^University of Miami School of Medicine, Schiff Center for Liver Diseases, USA; ^5^East China Normal University, Shanghai, China

The liver provides essential functions required to maintain homeostasis and the health of many organisms. During embryonic development, hepatocytes and biliary epithelial cells arise from the endoderm layer, whereas nonparenchymal liver cells emerge from the mesoderm layer. In a healthy adult liver, hepatocytes have a very slow turnover rate but they retain the capacity to rapidly repopulate the liver upon loss of liver mass [1]. Liver stem cells are called upon to regenerate the liver parenchyma when the ability of hepatocyte proliferation is compromised [2]. Over the years, a wealth of knowledge has accumulated pertaining to the cellular and molecular events of liver stem cell activation and differentiation in various animal models and human liver diseases. This knowledge is the driving force to study stem cells *in vitro* and apply them in laboratory and clinical practice.

Due to difficulties in isolating stem cells in adult livers, the exact origin of liver stem cells has not been resolved. It is widely believed that liver stem cells are located at the Hering canal, the conjunction between hepatocytes and the biliary tree. It is also postulated that the liver stem cells are among the nondescript cells in the periportal area. The hypotheses came from the observation that the progenies of putative liver stem cells, commonly known as oval cells, can be activated in various animal models such as dipin-induced mouse hepatocarcinogenesis or rats fed with 2-acetylaminofluorene in combination with partial hepatectomy. These oval cells phenotypically resemble the hepatoblasts in the embryonic liver and have the potential to differentiate into both mature hepatocytes and biliary epithelial cells. Recently, Yimlamai and Christodoulou et al. discovered in a mouse model that mature hepatocytes are highly plastic and can revert back (dedifferentiate) to a stem cell-like state [3]. Therefore, the liver seems to have the ability to regenerate itself via different mechanisms depending on the liver injury/stress that drives the need for new hepatocytes to be generated.

The development of induced pluripotent stem cells (iPSCs) and the differentiation of embryonic stem cells (ESC) and iPSC into various cell lineages *in vitro* over the last decade have greatly expanded our ability to model and treat various diseases. In particular, stem cell-derived hepatocytes have been used to model hepatotropic virus infection, virus and host interactions, and high-throughput drug screening, as well as hepatocyte transplantation in animal models [4]. Liver stem cells are also a promising tool in gene modification of genetic abnormalities, such as metabolic liver diseases and liver regeneration in cases of acute liver failure, cirrhosis, and hepatocellular carcinoma. In this special issue, Wang et al. reviewed the current state of cell culture models for various hepatitis viruses and highlight new and exciting models based on recent advances in stem cell technology.

In recent years, stem cell therapy solutions are being explored for the treatment of multiple liver diseases. In particular, mesenchymal stem cells (MSCs) are emerging as a useful tool for the treatment of liver cirrhosis. Patients with cirrhosis typically require liver transplantation following hepatic decompensation. More than one million patients around the world die each year while waiting for the transplants due to shortage of viable donor organs. Therefore, halting the progression of liver cirrhosis is a desirable approach for this deadly disease. MSC therapy has shown favorable results in improving the functions of the cirrhotic liver. In this issue, we are publishing several articles related to this topic. L. Chen et al. reported that the conditioned medium derived from mesenchymal stem cells could reduce liver cell damage *in vitro* and *in vivo*. Y. Zhang et al. revealed the therapeutic effect of human umbilical cord mesenchymal stem cells on acute liver failure in rats. X. Dong et al. characterized intestinal microecology during mesenchymal stem cell-based therapy for mouse acute liver injury. Y.-B. Pang et al. demonstrated a potential antitumor effect of dendritic cells fused with cancer stem cells in hepatocellular carcinoma. This issue also includes an article proposing a murine model for liver-directed cell transplantation which utilizes the spleen as a subcutaneous injection port (Miki et al.).

Despite the recent progress, great challenges remain. For example, the differentiation of stem cells into hepatic lineage is stalled at the stage of hepatocyte-like cells (HLCs) failing to achieve fully mature hepatocytes [4]. Further development of HLC into fully matured hepatocytes is required to reach the full potential of its research and clinical applications. Gene editing can cause off-target genetic alterations, a serious safety concern for clinical practice. Grafting of the liver stem cells or HLCs in animal models has had only measured success [5]. Overcoming these issues will get us closer to the reality of utilizing stem cells in the treatment of various liver diseases.

We hope this special issue provides a wealth of basic scientific data and introduces new concepts facilitating recent advances in the applications of stem cell technologies for the study of molecular mechanisms of hepatocyte growth and differentiation, pathogenesis, modeling, and liver disease therapies.
